# Super-Resolution Parameter Estimation Using Machine Learning-Assisted Spatial Mode Demultiplexing

**DOI:** 10.3390/s25175395

**Published:** 2025-09-01

**Authors:** David R. Gozzard, John S. Wallis, Alex M. Frost, Joshua J. Collier, Nicolas Maron, Benjamin P. Dix-Matthews, Kevin Vinsen

**Affiliations:** 1 International Centre for Radio Astronomy Research, The University of Western Australia, Crawley, WA 6009, Australia; john.wallis@research.uwa.edu.au (J.S.W.); alex.frost@research.uwa.edu.au (A.M.F.); joshua.collier@research.uwa.edu.au (J.J.C.); nicolas.maron@uwa.edu.au (N.M.); benjamin.dix-matthews@uwa.edu.au (B.P.D.-M.); kevin.vinsen@uwa.edu.au (K.V.); 2 Australian Research Council Centre of Excellence for Engineered Quantum Systems, Department of Physics, The University of Western Australia, Crawley, WA 6009, Australia

**Keywords:** quantum imaging, super-resolution, machine learning

## Abstract

We present the use of a light-weight machine learning (ML) model to estimate the separation and relative brightness of two incoherent light sources below the diffraction limit. We use a multi-planar light converter (MPLC) to implement spatial mode demultiplexing (SPADE) imaging. The ML model is trained, validated, and tested on data generated experimentally in the laboratory. The ML model accurately estimates the separation of the sources to up to two orders of magnitude below the diffraction limit when the sources are of comparable brightness, and provides accurate sub-diffraction separation resolution even when the sources differ in brightness by four orders of magnitude. The present results are limited by cross talk in the MPLC and support the potential use of ML-assisted SPADE for astronomical imaging below the diffraction limit.

## 1. Introduction

Classically, the angular resolution of an imaging system is limited by the diameter of the primary receiving optic and the wavelength of the received light according to Rayleigh’s criterion [1], and the similar Abbe and Sparrow criteria [2,3], which can be approximated as θ=λ/D, where θ is the smallest resolvable angular separation, λ is the wavelength of the light, and *D* is the diameter of the optic. Also known as the diffraction limit, this criterion motivates the construction of ever larger astronomical telescopes such as the Extremely Large Telescope [4] and the Square Kilometre Array [5].

However, the diffraction limit is not a fundamental limit, but a classical heuristic that only applies to pixel-by-pixel measurements of the intensity of the received field, which is referred to in the literature as direct imaging (DI). Quantum imaging methods motivated by advances in the understanding of quantum information have resulted in imaging systems that surpass the classical diffraction limit by utilizing quantum entanglement or other methods of engineering or controlling the source light, and have delivered outstanding results in fields such as microscopy and lithography [6]. However, in astronomical imaging, we have no way of controlling the source or probing the target object, and are restricted to the case of incoherent thermal radiation. In recent years, spatial mode demultiplexing (SPADE) has been shown to achieve quantum optimal performance [7,8,9,10,11], and numerous recent laboratory-scale experiments have demonstrated the ability of SPADE imaging to resolve sources orders of magnitude below the diffraction limit [12,13,14,15,16,17,18,19,20,21,22,23].

SPADE imaging works by decomposing the optical field into Hermite Gaussian (HG) modes and measuring the flux in each mode. This takes advantage of the sensitivity of the mode coupling to the offset of the wavefunctions from the centroid, which extracts additional phase information, providing super-resolution and beating the diffraction limit [7,8]. While there are many different ways to perform the mode sorting, many recent works [12,13,14,15,16,17,23] have focused on the use of multi-planar light converters (MPLCs) [24,25], also known as diffractive neural networks [26,27], due to the recent availability of the commercial-off-the-shelf (COTS) units with good reliability.

So far, laboratory demonstrations of SPADE imaging with MPLCs have used only the lower-order modes. Rouvière [14] and Santamaria [15,16] used only the HG_00_, HG_01_, and HG_10_ modes, while Wallis et al. extended the measurement to the HG_02_ and HG_20_ modes [23]. However, all of these works demonstrated determination of the relative separation and brightness of two objects, albeit achieving accurate measurements of these parameters down to separations on an order 100× lower than the diffraction limit. Parameter estimation in this simplified setup requires only comparing the flux in the non-zero HG modes to some reference, such as the HG_00_ mode.

As SPADE imaging demonstrations move towards using a greater number of modes, or towards the practical imaging of arbitrary source distributions, more advanced image reconstruction methods will be required. Moment-based measurements [28], iterative algorithms [29], and neural networks [30,31,32,33] have all been proposed as solutions to this problem. References [30,31,32] demonstrated the ability of SPADE plus neural network systems to classify letters or images from the Modified National Institute of Standards and Technology (MNIST) dataset despite severe blurring due to diffraction, while Sajia et al. [33] demonstrated the use of a physics-informed machine learning model trained from a simulated data set to determine the relative separation and brightness of two sources from a DI camera.

In this work, we experimentally demonstrate the use of machine learning to perform super-resolution source parameter estimation from an MPLC-based SPADE imaging system. The imaged sources and training data are generated physically in a laboratory. The resulting training, verification, and test data thus incorporate noise sources such as background noise, detector noise, optical losses, modal cross talk, centroid misalignment and other experimental factors. The resulting optical field is decomposed by a COTS MPLC with the flux from the outputs of four HG modes measured using single photon detectors. The training data is used to train a light-weight machine learning model that is small enough to be deployed on a COTS field-programmable gate array (FPGA) system, which would enable real-time measurement of the separation and relative brightness of the sources. The success of this work shows the potential for machine learning-assisted SPADE imaging systems to perform real-time super-resolution imaging, demonstrates what can be achieved with small ML models, and provides direction for scaling up such systems to use more modes with larger models to image more complicated sources.

## 2. Methods

### 2.1. Optical Setup

**Figure 1:**
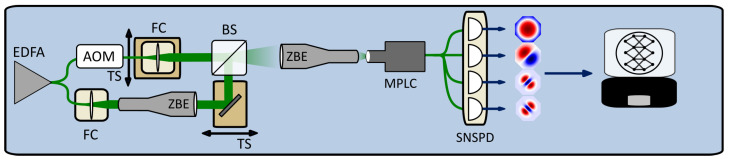
Simplified schematic of the machine learning-assisted SPADE imaging system. EDFA, erbium-doped fiber amplifier; AOM, acousto-optic modulator; BS, beam-splitter; TS, translation stage; FC; fiber collimator; ZBE, zoom beam expander; MPLC, multi-planar light converter; SNSPD, superconducting nanowire single photon detector. Each HG*_nm_* mode is individually coupled into a single mode fiber before single photon detection. For each detector, the photon count rate is logged with a time tagger.

A simplified schematic of the optical setup is shown in Figure 1. A broadband spontaneous emission of around 1550 nm from an unseeded erbium-doped fiber amplifier (EDFA) is split using a fiberized beam splitter and used to simulate two incoherent thermal sources. We used a Mach–Zehnder interferometer to determine that the coherence length of the EDFA light was less than 500 μm. The path length difference between the two sources is in the order of tens of centimeters, so there is no mutual coherence between the sources in this setup. An acousto-optic modulator (AOM) was used in one of the arms to attenuate one source (referred to as the secondary source) relative to the other (referred to as the primary source). We verified that the AOM introduced 1 dB of optical attenuation per 1 dB of radio frequency driving power over the 40 dB range of relative source powers used in this experiment. The EDFA’s output was globally attenuated to ensure the single photon detectors were not saturated.

The EDFA light is coupled from fiber into free space using two fiber couplers. Mirrors and motorized translation stages (TSs) are used to direct the beams to the two inputs of a beam splitter. The TSs allow the beams to be moved, to overlap them, or to separate them by some distance *d*. Two zoom beam expanders (ZBEs) are used to condition the beams to have the same size and focal point. The first beam expander is used to match the beam representing the primary source to the beam representing the secondary source. Each beam has a waist radius of $w_{0}$ = 1.5 mm at the beam splitter. The second beam expander is used to match the waist and focal points of the two beams to the input of a PROTEUS-C MPLC from Cailabs (Rennes, France). The alignment and coupling of the primary and secondary sources into the MPLC are adjusted using the ZBE and steering mirrors. The alignment is fine tuned by maximizing the isolation between the HG_00_ mode and the HG_01_ and HG_10_ modes [15,23]. This matches the Gaussian profile of the source to the HG_00_ mode of the MPLC. The combination of the SMF collimators and ZBE simulates an imaging system with a Gaussian point spread function (PSF). Astronomical telescopes have an Airy disc PSF, but a Gaussian PSF is a good approximation for the purposes of laboratory-scale demonstrations. The Cailabs PROTEUS-C MPLC has 10 outputs from modes between HG_00_ and HG_33_; however, we only had access to four superconducting nanowire single photon detectors (SNSPDs).

Real astronomical scenes have broad spectra and random polarization, and so it is important for the machine learning-assisted SPADE imaging system to handle these broad signals. The broadband spontaneous emission from the EDFA spans hundreds of nanometers, and is unpolarized, so effectively simulates the thermal emissions of stars. The MPLC is designed to operate across the whole International Telecommunications Union (ITU) C-band, so also has a broadband response suitable for processing star-like thermal signals in this band.

The MPLC is extremely sensitive to alignment due to the number of internal reflections inside the device [34]. We characterized the response of the modes by rastering one of the source beams over the input of the MPLC. We found that while the HG_01_, HG_02_, and HG_20_ modes matched theoretical curves for the expected mode response well [23], the HG_10_ differed significantly from the expected response. We measured the cross talk from the HG_00_ mode into the four higher-order modes and found that the HG_10_ mode had significantly greater cross talk of −9 dB, compared with the −21 dB to −25 dB of the other modes. Because of this, we excluded the HG_10_ mode and measured the outputs of the HG_00_, HG_01_, HG_02_, and HG_20_ modes on the SNSPDs. However, the effects of the poor HG_10_ mode could not be removed entirely because of the cross talk between the HG_10_ and HG_00_ modes. This leads to an increased noise floor from leakage photons and a deviation from the ideal response of the HG00 mode that diminishes the performance of the system. In addition, the HG_01_ and HG_10_ are the modes that are most sensitive to sub-diffraction separations, so the loss of reliable information from the HG_10_ mode is expected to diminish the performance of the system further.

SPADE imaging is extremely sensitive to the alignment of the optical center of mass (OCOM) of the target to the central axis of the imaging receiver [7,8,35,36]. Alignment errors beyond a few percent quickly degrade the accuracy of the imaging system [35,36] and it is not possible for a SPADE system to simultaneously estimate the OCOM, separation, and relative brightnesses of the sources [10,11]. However, measuring the OCOM has no equivalent to the diffraction limit, and accurate measurements of the OCOM can be made with DI [36]. We maintain the OCOM of the sources on the central axis of the MPLC by translating them to make them inversely proportional to their brightness. We used a Michelson interferometer to measure the repeatability of the TS to better than 5 μm. This is sufficiently accurate to allow us to use open loop control to adjust the separation of the sources based on their known relative powers. We verified that this procedure adequately maintained the OCOM by substituting a quadrant photodetector (QPD) for the MPLC and increasing the total power in the system to levels detectable by the QPD. Thus, the commanded position of the TSs is considered to be the ‘true’ separation in this experiment.

The experiment is performed by using the TS and AOM to sample the separation, *d*, and relative brightness, ϵ, parameter space. The TSs are used to generate 50 different separations logarithmically spaced between *d* = 20 μm (0.013$w_{0}$) and *d* = 6 mm (4$w_{0}$), while the AOM is used to attenuate the power of the secondary source relative to the primary between ϵ = 0 dB and ϵ = −40 dB in 2 dB increments. At each point, the number of photons measured by the SNSPDs is recorded over a 0.25 s integration time. Each point in this parameter space was sampled 120 times, creating a total data set of 120,000 data points that was used to train, validate, and test the machine learning model.

### 2.2. Machine Learning Model

We used the Moku Neural Network package [37] from Liquid Instruments (Canberra, Australia) to train an artificial neural network (ANN) to predict the separation, *d*, and relative brightness, ϵ, of the imaged sources from the measured photon number in the HG_00_, HG_01_, HG_02_, and HG_20_ modes measured at the SNSPDs. Liquid Instruments supply FPGA-based test and measurement equipment, including the Moku:Pro, which features a Xilinx Ultrascale+ FPGA chip. The Moku Neural Network package uses the TensorFlow [38] implementation of Keras to train and deploy ANN models on the Moku:Pro’s FPGA. Moku Neural Network is user-friendly software for the training of ANNs and, in principle, allows for easy deployment onto the Moku:Pro hardware. The advantage of this system is that the Moku:Pro is capable of directly reading in the counts from the four SNSPDs, scaling the signals, and then outputting the separation and relative brightness values in real time, all on a single device. The Moku:Pro is only able to support small ANNs of up to a maximum of five layers, each with up to 100 neurons. The ability to deploy such a ‘light-weight’ ANN model on a versatile measurement device and output the desired values in real time demonstrates the practicality and versatility of this technique.

For each of the data points obtained during the experimental data acquisition, the data are conditioned by first taking the ratio of the HG_01_, HG_02_, and HG_20_ modes to the HG_00_ mode. This prevents the total brightness of the source from impacting the model’s learning. The count values range over several orders of magnitude, so we take the logarithm of the ratios before normalizing the data to range between −1 and 1. We also take the logarithm of the separation values, which ranged over two orders of magnitude, before normalizing them. The relative brightness values, which ranged from 0 dB to −40 dB, are already in log space, so only required normalization.

Since we have taken the ratio of the HG_01_, HG_02_, and HG_20_ modes to the HG_00_ mode, this means there are three input values for each separation and relative brightness data point to feed into the model. The ANN model used in this work comprises five dense layers of 64, 32, 32, 8, and 2 neurons, respectively. All layers use a hyperbolic tangent (tanh) activation function. The model outputs two values, the first being the separation of the sources, and the second being the relative brightness. These values then needed to be denormalized from the −1 to 1 output values to the true separation and relative brightness values.

Of the 120,000 data points, 80% were used for training, 10% for validation, and 10% for testing. The validation and test data were extracted with a uniform random distribution. The model was trained with an early stopping configuration, and would typically train for 150 to 250 epochs, taking around 30 min on an NVIDIA GeForce RTX 4060 Laptop GPU. Mean squared error was chosen for the loss function.

## 3. Results

**Figure 2:**
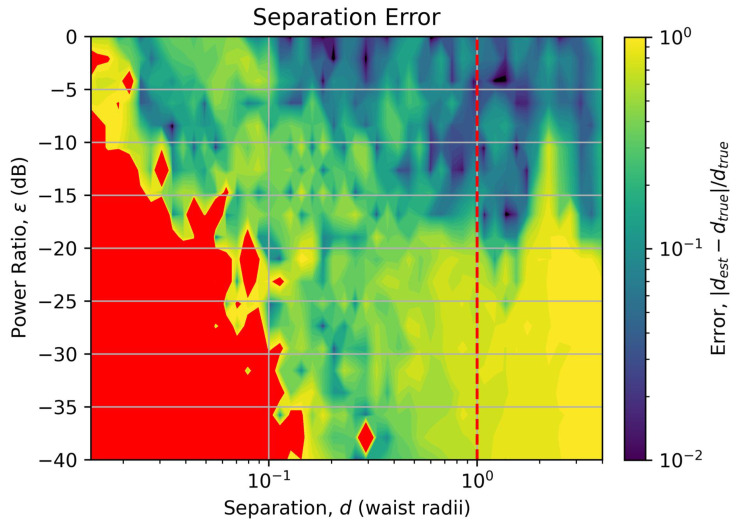
Error in the source separation estimation over the range of true source separations (x-axis) and relative source powers (y-axis). Points with errors exceeding 100% have been colored red. Points with errors less than 1% have been colored black. The dashed red line indicates the diffraction limit.

**Figure 3:**
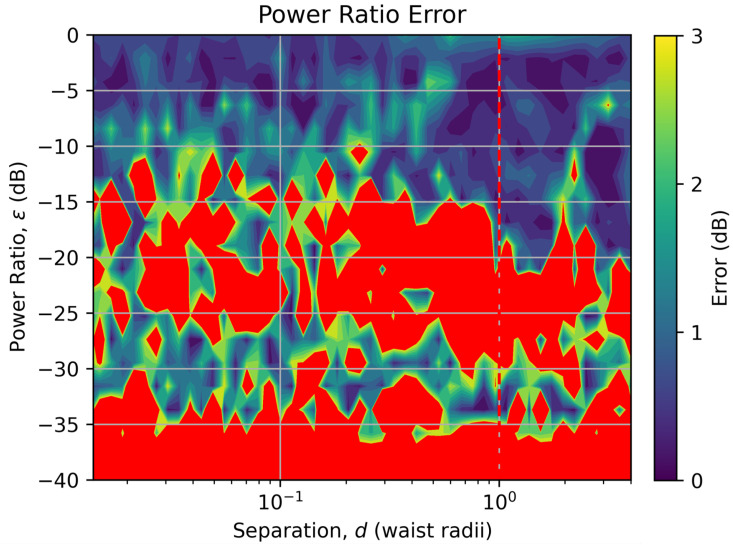
Error in the source relative power estimation over the range of true source separations (x-axis) and relative source powers (y-axis). Points with errors exceeding 100% have been colored red. The dashed red line indicates the diffraction limit.

Following training of the model, the random 12,000 test data points were then used to assess the performance of the model over the separation and relative brightness parameter space. Figure 2 shows the error in the separation estimation, while Figure 3 shows the error in the power estimation.

In Figure 2, the separation error is shown as a color map, with the error value displayed as a fraction of the true separation according to $|\left(d_{estimated}-d_{true}\right)|/d_{true}$. Regions where the error is equal to or exceeds 100% (i.e., a factor of two) are colored red. Regions where the error is less than 1% are colored black. Restricting the color map shading to logarithmically spaced values between 1% and 100% errors helps to highlight features in the plot. The vertical dashed red line indicates the diffraction limit equivalent to θ=λ/D for this laboratory-scale system.

In Figure 3, the error in the power estimation is shown as a color map with the error value displayed as the absolute difference between the true and estimated relative power in dB. Regions where the error is equal to or exceeds 3 dB (a factor of two) are colored red. Again, the vertical dashed red line indicates the diffraction limit equivalent to θ=λ/D for this laboratory-scale system.

## 4. Discussion

From Figure 2, it can be seen that the model accurately estimates the separation of the sources down to nearly two orders of magnitude below the diffraction limit when the sources are of reasonably equal brightness. As the difference in power between the two sources increases, the accuracy of the estimation reduces, but the separation estimate still remains accurate an order of magnitude below the diffraction limit.

Figure 3 shows that the power ratio estimation is good when the relative powers differ by less than a factor of 10; however, the accuracy of the relative power estimation over the rest of the parameter space is very poor, with estimates for sources with power ratios below −10 dB becoming very unreliable. The band of, what appears to be, quite accurate power ratio estimates around −30 dB, are due to the fact that below about −20 dB, the model estimates approximately the same relative power value for all separations and relative powers, which coincidentally clusters around an estimate of −30 dB, giving a false indication of accuracy if Figure 3 alone is taken at face value.

The degradation in performance of the separation estimation, and very poor performance of the power ratio estimation for sources with power ratios below −10 dB, is attributed to the poor coupling and cross talk of the HG_10_ mode. Not only do we, as noted previously, lose critical information about very small separations, the significantly higher cross talk between the HG_10_ and HG_00_ modes compared with the other modes prevents accurate measurements of the flux in the HG_00_ mode, which is crucial when most of the light is coupling into the HG_00_ mode and very little into the higher-order modes, as is the case at these large power differences and small separations. The region in the bottom-right of Figure 2, where the separation error is approaching 100% even though the source separation is greater than the diffraction limit, is attributed to cross talk from the HG_10_ mode into the HG_00_ mode creating an anomalously large signal in the HG_00_ mode, making the model think that the sources are closer than they really are.

This result highlights the importance of the quality of the MPLC to the parameter estimation. Low cross talk between modes is crucial to improving the performance of this form of SPADE imaging, and to push the accurate performance of the system to smaller separations and greater power differences, as would be needed for realistic applications in astronomy. Increasing the size of the ANN model, or changing the shape of the model, within the Moku:Pro’s limit of five layers with up to 100, neurons did not significantly improve the separation or relative power estimation.

Similar MPLC-based SPADE imaging systems (for example, [15,16]) have used only the HG_01_ and/or HG_10_ modes to extract imaging information. The use of only these lower-order modes leads to a degeneracy in the response of the MPLC, where the SPADE system is unable to discern the difference between a bright secondary source at low separation and a dim secondary source at larger separation [23]. The purpose of including the higher-order HG_02_ and HG_20_ modes is to break this degeneracy and enable simultaneous estimation of the separation and relative power parameters. The results show that this has been broadly successful, with the ANN model demonstrating accurate separation and power estimation over the regions of the parameter space where the cross talk from the HG_10_ mode does not significantly degrade the measurement. However, follow-up work using an MPLC with a less noisy HG_10_ mode will be needed to accurately evaluate the performance of this SPADE imaging method over the full parameter space measured in this experiment.

The performance of the machine learning-assisted SPADE imaging system is almost the same as the performance of the same system using maximum likelihood estimation presented in reference [23]. We do not expect a significant improvement in performance over [23] using machine learning in this work due to the simple image source and small number of modes being measured. The present work presents a preliminary proof-of-concept experiment showing no barriers to the expansion of this machine learning-assisted SPADE experiment in the future.

## 5. Conclusions

We have demonstrated ML-assisted super-resolution estimation of the separation and relative brightness of two incoherent light sources. This is the first experiment, to our knowledge, that demonstrates ML-based SPADE imaging using training data derived from laboratory measurements and which uses laboratory measurements to evaluate the performance of the model.

The MPLC-based SPADE system and ML model accurately estimated the separation and relative brightness of the sources down to two orders of magnitude below the classical diffraction limit when the sources were of roughly equal brightness, and were able to accurately estimate the separation of the sources down to one order of magnitude better than the diffraction limit when the power of the sources differed by as much as 40 dB (a factor of 10,000) despite the negative impact of excessive cross talk in one mode of the MPLC.

The present work represents a preliminary demonstration of the ML-assisted SPADE technique. The training set is relatively small (96,000), and the validation and analysis data only revisited points in the parameter space that the ANN model had seen during training. A larger training set will improve the training of the model. This larger training set will also benefit from sampling the parameter space more thoroughly. Testing the model and analysis of its performance will also be improved by testing the model on data points with parameters in between the points used in the training data to evaluate the ability of the model to interpolate between points used in the training.

The model trained in this work is designed to be deployed on a Moku:Pro FPGA. Doing so will allow us to demonstrate the ability of the model to provide real-time estimation of the source parameters without offloading the SNSPD signals from the Moku:Pro to a computer. The latency of this ANN model on the Moku:Pro is 154 clock cycles, or approximately 0.1 μs.

While the present work only investigated two sources translating in one dimension, the use of HG modes separated by 90° means that this SPADE system should be able to measure sources with an unknown two-dimensional distribution. This may be possible with the limited size of the ML model allowed by the Moku:Pro; however, as more complicated sources are imaged, the size of the model required to accurately estimate the brightness and distribution of the sources will increase, and it is likely that the Moku Neural Network will not be large enough to estimate sources much more complicated than the simple two-source case in the present work. The Moku Neural Network is only capable of supporting four input values, so any SPADE system measuring more than four modes will need to use an alternative platform.

Regardless of whether Moku Neural Network, another ML platform, or a different algorithm entirely is used to reconstruct the imaged field from the outputs of the MPLC, this work shows that the quality of the MPLC, in particular, the cross talk between the modes, is crucial in creating an effective imaging system. For an astronomy application such as the detection of exoplanets, the SPADE system will need to be able to discern the presence of a second object around eight orders of magnitude dimmer than its host star [39]. MPLCs with significantly better isolation between modes will be needed to achieve this goal.

MPLCs have a strong wavelength dependence. The Cailabs PROTEUS-C used in the present work is designed to work across the ITU C-band (approximately 1520 nm to 1577 nm), which sits within the astronomical H-band (approximately 1476 nm to 1784 nm). Using an MPLC to efficiently image broad spectrum (stellar) sources while achieving very high isolation between modes is a technical challenge that requires further investigation. As noted in the experimental description, the MPLC is extremely sensitive to alignment. Future work will also need to investigate the impact of telescope tracking precision on practical imaging and methods to mitigate this as a source of error.

## Data Availability

Data supporting this study can be obtained from the corresponding author upon reasonable request.

## Abbreviations

The following abbreviations are used in this manuscript:

ANN	artificial neural network
AOM	acousto-optic modulator
BS	beam-splitter
COTS	commercial-off-the-shelf
DI	direct imaging
EDFA	erbium-doped fiber amplifier
FC	fiber collimator
FPGA	field programmable gate array
GPU	graphics processing unit
HG	Hermite Gaissian
ITU	International Telecommunications Union
ML	machine learning
MNIST	modified National Institute of Standards and Technology
MPLC	multi-planar light converter
OCOM	optical center of mass
PSF	point spread function
QPD	quadrant photodetector
SMF	single-mode fiber
SNSPD	superconducting single photon detector
SPADE	spatial mode demultiplexing
TS	translation stage
ZBE	zoom beam expander
